# Eosinophil extracellular traps in asthma: implications for pathogenesis and therapy

**DOI:** 10.1186/s12931-023-02504-4

**Published:** 2023-09-26

**Authors:** Kunlu Shen, Mengyuan Zhang, Ruiheng Zhao, Yun Li, Chunxiao Li, Xin Hou, Bingqing Sun, Bowen Liu, Min Xiang, Jiangtao Lin

**Affiliations:** 1 National Center for Respiratory Medicine, National Clinical Research Center for Respiratory Diseases, Institute of Respiratory Medicine, Department of Pulmonary and Critical Care Medicine, Center of Respiratory Medicine, Chinese Academy of Medical Sciences, Friendship Hospital, No.2, East Yinghua Road, Chaoyang District, 100029 Beijing, China; 2 Peking Union Medical College, Chinese Academy of Medical Sciences, Beijing, China; 3 Beijing University of Chinese Medicine, Beijing, China; 4 Peking University Health Science Center, Beijing, China

**Keywords:** Asthma, Eosinophil extracellular traps, EETs, Eosinophil extracellular trap cell death, Therapeutics, Anti-EETs

## Abstract

Asthma is a common, chronic inflammatory disease of the airways that affects millions of people worldwide and is associated with significant healthcare costs. Eosinophils, a type of immune cell, play a critical role in the development and progression of asthma. Eosinophil extracellular traps (EETs) are reticular structures composed of DNA, histones, and granulins that eosinophils form and release into the extracellular space as part of the innate immune response. EETs have a protective effect by limiting the migration of pathogens and antimicrobial activity to a controlled range. However, chronic inflammation can lead to the overproduction of EETs, which can trigger and exacerbate allergic asthma. In this review, we examine the role of EETs in asthma.

## Background

Asthma is a chronic inflammatory airway disease in which neutrophils and eosinophils play an important role [1, 2]. Activated neutrophils can release granules through degranulation, internalize and degrade pathogens through phagocytosis, and release neutrophil extracellular traps (NETs) to defend against external pathogens [3]. Studies have shown that excessive levels of NETs can damage the airway epithelium and trigger an inflammatory response, increasing the severity of asthma [4].

Eosinophils are known for their role in asthma as end-effector cells in allergic diseases [5]. Upon receiving stimulatory signals, they undergo tissue migration and perform immunomodulatory and pro-inflammatory functions by releasing immunomodulatory factors (cytokines, chemokines, growth factors), and cytotoxic proteins that are pre-formed and stored intracellularly [1, 6].

**Fig. 1:**
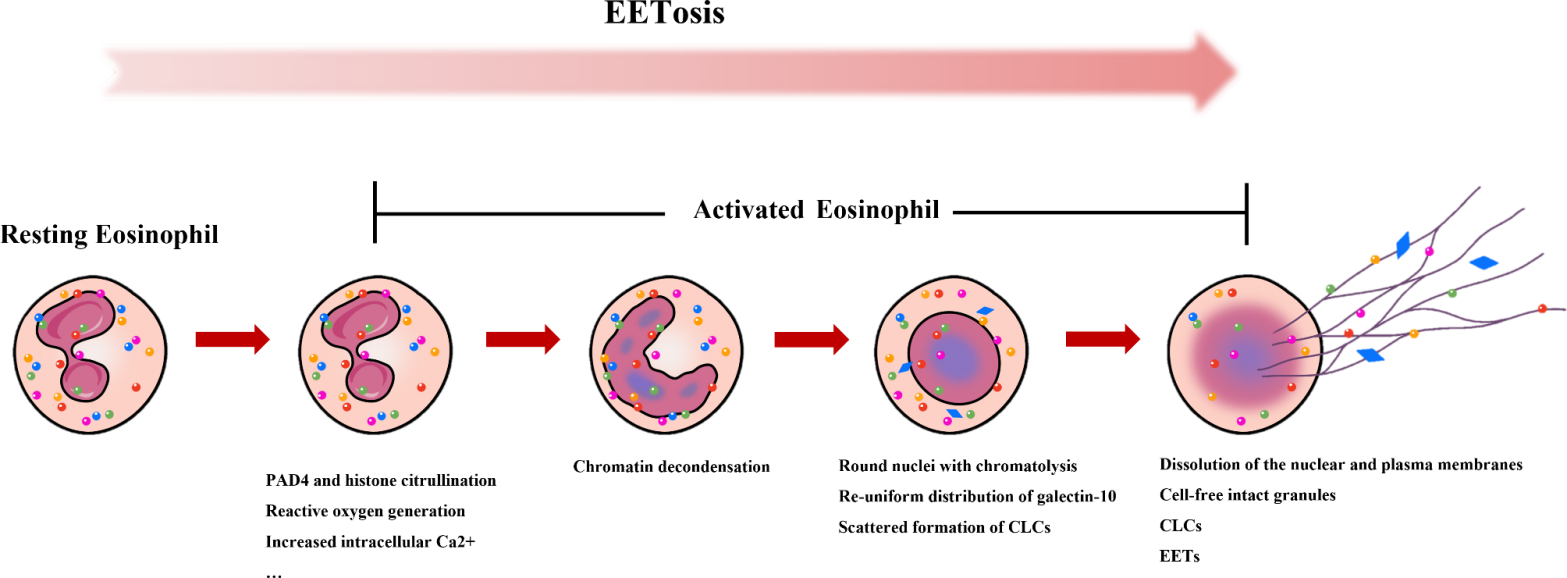
**Time course of EETosis.** Many ex vivo and in vivo stimuli can lead to human eosinophil activation, increased intracellular Ca2 + and reactive oxygen species production, PAD4-mediated citrullination of nucleosomal histones, resulting in chromatin decondensation with nuclear rounding. Additionally, there is galectin-10 redistribution and occasional formation of intracellular CLCs prior to plasma membrane lysis. Finally, both nuclear and plasma membranes are disassembled, and reticulated chromatin structures are released. EETosis: Eosinophil extracellular trap cell death; PAD4: Peptidyl arginine deaminase 4; CLCs: Charcot-Leyden crystals; EETs: Eosinophil extracellular traps

In addition, recent studies have observed that eosinophils can form extracellular traps similarly to neutrophils. By releasing nuclear DNA into an extracellular backbone and embedding granule proteins in this backbone, a new cell death pathway distinct from necrosis and apoptosis is formed, called eosinophil extracellular trap cell death (EETosis; Fig. 1) [7]. A variety of microorganisms and their products, as well as non-infectious stimuli, can activate this pathway, which is regulated by multiple activation mechanisms, including toll-like receptors, chemokines, cytokines, and adhesion receptors. These mechanisms can initiate transmembrane signaling leading to the formation of eosinophil extracellular traps (EETs) [6, 8].

Like NETs, EETs are important for host defense against extracellular pathogens by providing scaffolding structures that offer attachment points for agents such as cytotoxic proteins, limiting the immune response to an effective and safe area [1]. However, unlike NETs, the chromatin structure on EETs is more stable and concentrated, and EETs are less susceptible to proteolytic degradation, allowing them to remain in the body for a longer period of time [9]. In chronic and uncontrolled asthma, overproduction of EETs can interact with other immune cells and exacerbate the inflammatory response [6, 9]. Studies have shown that EETs are elevated in asthmatic patients and correlate with the severity of the disease [10]. In ovalbumin (OVA)-challenged mice, increased EETs secretion has been associated with greater cellular infiltration, airway inflammation, and mucus secretion [11]. Additionally, it has been shown that EETs may be associated with a failure to respond to steroid hormone therapy for asthma [12].

Although the exact mechanisms by which EETs contribute to chronic inflammatory airway disease remain unknown, the importance of studying EETs should not be underestimated. A deeper understanding of EETs may provide new insights for the development of biomarkers to aid in the diagnosis of asthma type and severity, as well as potential new targets for treatment. This review provides a summary of the general characteristics of EETs, their relevance to asthma, and potential mechanisms of action and therapeutic targets in the development of asthma.

## Eosinophils and degranulation

Degranulation is a critical function of eosinophils associated with many allergic and inflammatory diseases [13]. It refers to the process by which living or dead cells release intact or ruptured specific granules [6]. These granules, also known as secondary or secretory granules, are membrane-surrounded structures that contain a dense crystalline core and a matrix containing various mediators that can induce inflammation and/or tissue damage. These mediators include basic proteins, cytokines, chemokines, growth factors, and enzymes, with proteins being the most important [14, 15].

Eosinophils contain both enzymatic and non-enzymatic cationic proteins in specific granules that are selectively secreted in response to stimulation [16]. These eosinophil-derived granule proteins (EDGPs) include eosinophil peroxidase (EPO), major basic protein (MBP), eosinophil cationic protein (ECP), and eosinophil-derived neurotoxin (EDN) [17]. These proteins have potent cytotoxic effects and may act by disrupting lipid bilayer integrity [18], exhibiting neurotoxicity and RNase activity, and/or participating in the generation of reactive oxidants and free radicals [19]. In addition to their cytotoxicity, EDGPs also play a critical role in eosinophil development [20]. Studies have shown that the simultaneous absence of MBP-1 and EPO can result in a loss of eosinophil precursors in the bone marrow as well as eosinophils in the peripheral blood [21].

In addition, activated eosinophils are capable of releasing galectin-10 (Gal10) and undergoing a phase transition to form Charcot-Leyden crystals (CLCs). These crystals persist in the tissues for several months and contribute further to the progression of asthma [22]. A study conducted by Persson et al. discovered that biomimetic crystals of Gal10 elicited an innate immune response rich in neutrophils and monocytes upon injection into the airways of young rats. Furthermore, when combined with OVA, it not only induced dendritic cell uptake and initiated type 2 helper T cell response but also resulted in an increase in airway eosinophils and an immunoglobulins (Ig) G1 response [23].

Eosinophils have multiple degranulation mechanisms, and the classical modes of degranulation include cytosolic spitting and progressive degranulation (also known as lamellar degranulation or segmental degranulation). Cytosolic degranulation refers to the fusion of the entire granule content with the plasma membrane as a separate granule, resulting in the release of the entire granule content, whereas progressive degranulation refers to the gradual and selective release of the granule content by small vesicles without fusion with other granules or the plasma membrane [13, 24, 25]. In addition, a unique degranulation pattern has been observed in eosinophils that were previously thought to be a form of “necrosis” [9, 26]. However, subsequent research has shown that this degranulation pattern does not exhibit the usual features of necrosis or apoptosis, such as phosphatidylserine expression (as shown by weak Annexin V staining) or necrotic spots, and represents a distinct mode of cell death called extracellular trap cell death (ETosis) [6, 27–29]. None of the known types of necrosis or extrusion artifacts could explain the mechanism of this degranulation pattern [6, 9].

The discovery of extracellular traps (ETs) dates back to 1975 when Anker et al. demonstrated in vitro that lymphocytes can release DNA into the extracellular space without compromising their activity [30]. In 2004, Brinkmann et al. observed that in addition to phagocytosis and degranulation mechanisms, neutrophils can kill bacteria in the extracellular space by releasing nuclear components that form extracellular fibers with granules, a phenomenon known as NETs [31]. Subsequently, in 2008, Yousefi et al. discovered that eosinophils can also exert their effects by releasing granule proteins and DNA to form extracellular structures later identified as EETs [32].

## Eosinophil extracellular trap death

ETosis is considered a potential type of cell death that promotes inflammation [33]. Eosinophils can be further classified into vital EETosis and suicidal EETosis depending on whether they undergo cell death [34]. It has been observed that eosinophils can release DNA from mitochondria by jetting, and after forming EETs, eosinophils remain viable without evidence of apoptosis or other types of cell death, a process known as vital EETosis [32].

For suicidal EETosis, the process is more complex and involves a series of morphological changes: disassembly of nuclear chromatin followed by rupture of the cytoplasmic membrane, which in turn leads to the release of EDGPs and nuclear material (e.g. histones and DNA) from the ruptured eosinophils into the surrounding environment and the production of EETs [35–37]. Although the above appears to be a continuous process, it has been observed to occur rapidly in vitro experiments (~ 0.5- 3 h), which may lead to a rapid release of nuclear chromatin and EDGPs after plasma membrane rupture without sufficient mixing, resulting in a detectable extracellular network of DNA fibers with or without intact EDGPs [9, 38]. In the case of suicidal EETosis, it somehow becomes a continuation of the eosinophil function and extends the function of eosinophils[39].

It has been suggested that the decision of whether EETosis is released in a viable or dead form may depend on the source of stimulation in vivo and in vitro [37], but there is still academic debate as to whether mitochondrial-derived DNA alone is sufficient to form such a large number of EETs upon stimulation [39]. The prevailing view is that the DNA for EETosis is nuclear in origin and that the cell must eventually release it through cell death formation [8]. There are three main reasons for this. First, the release of DNA from the mitochondria into the cytoplasm and then into the extracellular compartment requires very high energy; second, a single eosinophil contains a small number of mitochondria with insufficient copies of DNA and is susceptible to structural damage caused by reactive oxygen species (ROS); and finally, mitochondrial DNA is stickier and smaller than nuclear DNA, making it easier to detect, which may partially explain the previous findings [6].

In allergic tissues, cytolysis is the second most common mode of degranulation after lamellar degranulation, accounting for 10–33% of all degranulation patterns [40–42]. Some studies suggest that this percentage may be as high as 80% in patients with eosinophilic esophagitis [43]. ETosis may also be present at high levels in severe and fatal asthma [26].

In isolated eosinophils, the induction of stimulated production of EETs occurs in a variety of ways, such that transmembrane signaling may be initiated first by cytokines (e.g., interleukin 5 [IL-5], interferon-γ), chemokines, or adhesion molecules, followed by lipopolysaccharide (LPS), eosinophil chemokine, or complement component 5a (C5a), thymic stromal lymphopoietin (TSLP), or staphylococcus aureus stimulation [8, 32, 44, 45]. In addition, it can also be induced by direct stimulation with sorbitol acetate, calcium ion carriers, immobilized IgA or IgG, phorbol 12-myristate 13-acetate (PMA), respiratory syncytial virus, and the calcium ionophore A23187 [38, 46, 47].

## Mechanism of action of EETs in asthma

### Airway epithelial cells and EETs

Airway epithelial cells are critical components of the innate immune system, contributing to the maintenance of airway structural integrity, barrier function, and ciliary clearance. However, in asthmatics, airway epithelial dysfunction increases susceptibility to viral infections [48, 49]. In addition, epithelial cells can perpetuate type 2 inflammation by releasing various cytokines, chemokines, and alarmins (such as IL-33, TSLP, and Granulocyte-macrophage colony-stimulating factor [GM-CSF]) and interacting with other immune cells, ultimately leading to airway wall remodeling in asthma [12, 50]. EETs have been shown to induce epithelial cell desquamation and increased permeability in a dose-dependent manner and to significantly enhance the release of IL-6 and IL-8 from epithelial cells [51]. When EETs were exogenously injected into a mouse model of asthma, epithelium-derived cytokine levels (including IL-1α, IL-1β, Chemokine ligand 1 [CXCL-1], CCL24, IL-33, and TSLP) were found to be significantly increased in mouse bronchoalveolar lavage fluid (BALF) [11]. A recent study suggests that TSLP may play a greater role than other allergen-induced cytokines in the induction of EETs formation [10].

### Epithelial cell-derived autoantibodies and EETs

Epithelial cell-derived autoantibodies, such as serum cytokeratin 18 (CK18), can also interact with EETs. CK18 are elevated in patients with severe asthma and are positively associated with total eosinophils and negatively correlated with forced expiratory volume in one second percentage (FEV1%). The presence of CK18-specific IgG induces degranulation of peripheral blood eosinophils in asthmatic patients, promoting the release of EETs, which could further stimulate the release of CK18 from airway epithelial cells, creating a vicious cycle. This phenomenon was also observed in asthmatic mice, and dexamethasone treatment did not effectively prevent it. Therefore, the immune response involving EETs and epithelial autoantigens may contribute to steroid-naive severe asthma, and targeting EETs may be a potential therapeutic strategy for severe asthma[52, 53].

### Pulmonary neuroendocrine cells and EETs

EETs have been identified as playing a critical role in the crosstalk between immune and neural signals that contribute to the development of severe asthma. Pulmonary neuroendocrine cells are rare multifunctional epithelial cells that account for approximately 0.4% of the total airway epithelium and are mainly located at the junction of airway branches [54]. Recent research has revealed that EETs activate pulmonary neuroendocrine cells through the CCDC25-ILK-PKCα-CRTC1 pathway, resulting in the secretion of neuropeptides and neurotransmitters (such as calcitonin gene-related peptide and gamma-aminobutyric acid). This, in turn, leads to the recruitment and activation of inflammatory immune cells and promotes mucus secretion from cupped cells, ultimately amplifying the immune response in asthma [10].

### Group 2 innate lymphoid cells (ILC2) and EETs

ILC2 are involved in not only mediating the activation of eosinophils in the asthmatic airways but also the development of steroid resistance. Recent studies have demonstrated that EETs can interact with ILC2 to further activate the innate immune response, exacerbating type 2 inflammation [11].

### Autocrine function of EETs

In addition to interacting with other immune cells, EETs have been shown to significantly increase the level of eosinophil degranulation and ROS production, suggesting that EETs have autocrine and immune response-inducing functions. This creates a vicious cycle that plays a key role in eosinophilic airway inflammation [51].

### Charcot-Leyden crystals and EETs

The formation of EETs is closely related to the production of CLCs. Both EETs and CLCs significantly affect the rheology of mucus, making it more difficult to clear from the airways [1, 23]. During EETosis, Gal10 is redistributed within the eosinophil cytoplasm, leading to the formation of intracellular CLCs, which are then released extracellularly. CLCs activate macrophages and epithelial cells, promoting the release of inflammatory cytokines and enhancing innate and adaptive immunity. Additionally, CLCs can promote type 2 immunity by increasing the production of the IgG1 and IgE [22, 23].

### Double-stranded DNA (dsDNA) and EETs

Respiratory viral infections are a major cause of acute exacerbations of asthma and are associated with the exacerbation of type 2 immune responses. Previous research has demonstrated that dsDNA functions as an endogenous danger signal or damage-associated molecular pattern, activating various pattern recognition receptor signaling pathways to alert the innate immune system [55]. Toussaint et al. showed that NETs and dsDNA play a key role in virus-induced asthma exacerbation [56]. Respiratory viral infection significantly contributes to dsDNA accumulation and enhances type 2 cytokine release, leading to the exacerbation of asthma-like symptoms in mice. Furthermore, by using eosinophil-deficient ΔdblGATA mice, the researchers confirmed that dsDNA was primarily derived from the release of neutrophil NETs and not from eosinophils [57]. In contrast, a study by Silveira et al. found that in vitro respiratory syncytial virus induced the formation of EETs in eosinophils from asthmatic mice [47]. The role of EETs in the acute exacerbation of virus-induced asthma is not fully understood, and further studies are needed to explore this.

## EETs Associated with severe asthma

Neutrophil-released NETs have been extensively studied for their role in infectious diseases. Eosinophils are considered to be one of the most important central effector cells in the development of asthma. In contrast to neutrophil phagocytosis, eosinophils promote airway inflammation and remodeling primarily through the selective release of various molecules such as cytokines and granulins [58, 59].

In non-infectious asthma, the production of EETs is significantly increased, contributing to the exacerbation of type 2 immunity. In OVA-challenged mice, both eosinophil and neutrophil extracellular traps were generated and released after allergen stimulation, with significantly higher levels of EETs production than NETs (57.09 ± 10.23% vs. 5.27 ± 3.32%) [10]. Intranasal administration of EETs for five days increased the number of eosinophils and neutrophils in the BALF of wild-type BALB/c mice, along with elevated levels of epithelial-derived cytokines (such as IL-1α, IL-1β, CXCL-1, CCL24, IL-33, and TSLP) and an increased proportion of IL-5 or IL-13-producing ILC2 cells in the lungs [11].

Moreover, several studies have shown that EETs may serve as potential biomarkers of severe asthma and are associated with acute exacerbations of asthma [59, 60]. In humans, levels of EETs in BALF were significantly higher in adult asthmatics than in healthy controls and were positively correlated with levels of type 2 cytokines (i.e., IL-4, IL-5, and IL-13) and asthma severity, while negatively correlated with FEV1% [10]. The study by Choi and colleagues demonstrated that severe asthmatics have a higher release of EETs compared to non-severe asthmatics when peripheral blood eosinophils are stimulated by IL-5 and LPS [51]. The study also showed a negative correlation between EETs and FEV1, and a positive correlation between EETs and serum eosinophil-derived neurotoxin levels. Follow-up studies by Lee et al. and Choi et al. confirmed these findings and further suggested that EETs production is associated with the degree of airway inflammation and obstruction in patients with severe asthma [11, 53]. However, a study by Granger et al. found no association between serum EETs and asthma severity, and EETs expression was not detectable in BALF [61]. Instead, this study showed that only serum NETs levels correlated with asthma severity, control level, age at onset and duration of asthma exacerbation.

Several factors contribute to the variability in the results of studies investigating ETs in asthma. First, the heterogeneity of the populations included in the studies may partially explain the observed differences. In mouse models of asthma following viral infection, an increase in neutrophil generation with NETs has been observed, peaking within 2 days [56, 62]. Respiratory infections are important triggers of asthma exacerbations, which tend to be more severe and have different pathological manifestations compared to non-infected asthma. Secondly, unlike in vitro experiments, the formation of ETs in vivo is influenced by various factors such as the type of stimulus, dose, exposure time, and drug treatment. Moreover, the rapid formation of ETs themselves may pose challenges in their in vivo detection [59, 63]. As noted by Granger et al., the expression of EETs in BALF was not effectively detected, even though 53% of patients with available BALF samples had alveolar eosinophil counts > 1.2% [61]. Therefore, future studies are needed to determine the impact of EETs expression levels on different asthma severities and sample sources.

## EETs as biomarkers

EETs can be monitored by a variety of means, but like NETs, they serve as screening tools to help clinicians better individualize treatment. This requires readily available test samples and tests that demonstrate good specificity, sensitivity, and reproducibility. Such tests should provide value for the diagnosis, treatment and prognostic monitoring of clinical disease.

As mentioned above, EETs are composed of several components. The DNA and citrullinated histone H3 (citH3), which form the “backbone”, and the granular proteins such as ECP and EPO, which are attached to the “backbone”, can be used as indicators of their levels. However, it should be noted that some markers are not only found in eosinophils with EETosis but can also be released by apoptotic or necrotic eosinophils and other cells, so it is worth considering how to combine and detect these markers. Visualization of EETs production by cellular immunofluorescence or immunohistochemistry is the most accurate method [64–66], but this is relatively inaccessible in asthmatic patients because it requires obtaining BALF and lung tissue samples in an invasive manner, such as bronchoscopy.

This led us to look at the more non-invasive and readily available blood samples. The detection of circulating free DNA (cfDNA) is one of the indicators of response to EETs that was first described in a study by Yousefi et al. [32]. In patients with eosinophilia with polyangiitis (EGPA), it was observed that cfDNA levels were significantly higher in EGPA patients than in healthy subjects and correlated significantly with disease activity and blood eosinophil count, and that the presence of EETs increased cfDNA levels in EGPA [67]. Circulating citH3 is another indicator that can be used as a response to EETs and shows better specificity than cfDNA [10], which can be used as a potential assessment indicator for loss of asthma control [68]. However, it is important to note that for in vivo detection of EETs, other innate immune cells besides eosinophils can also release ETs [69], a process that may also involve citrullination of histones, a point that has been well-documented for NETs [3, 70]. Compared to circulating cfDNA and citH3, a modified ELISA targeting the ECP-DNA complexes characteristic of EETs may be more specific [10, 69, 70].

In addition, several novel biomarkers have been proposed that may reflect the levels of EETs. In patients with chronic rhinosinusitis (CRS), calmodulin was found to be expressed in eosinophils and involved in the innate immune response via EETs, which could be used as a biomarker in response to the severity of CRS [71], but it is unclear whether it could be used as one of the indicators of EETs levels in asthma. Gal10 is an eosinophil-specific marker released by EETosis, and elevated levels of Gal10 in serum and tissues may indicate an increase in the number of eosinophils that undergo EETosis in vivo. Further future studies are needed to clarify the scenario and value of these biomarkers in asthma patients.

## Therapeutic targets for EETs

The advent of biological agents has opened new avenues for the treatment of patients with severe asthma. However, there remains a subset of patients with a limited response to corticosteroids or biologic agents, highlighting the need for the identification of new therapeutic targets and biomarkers. The therapeutic targets for EETs can be approached from two perspectives: a pre-EET formation block, in which eosinophils receive various signaling stimuli in vivo to initiate the EETosis process, and a post-EET release block, which includes targeting the chromatin backbone structure that constitutes EETs and substances attached to chromatin.

**Fig. 2:**
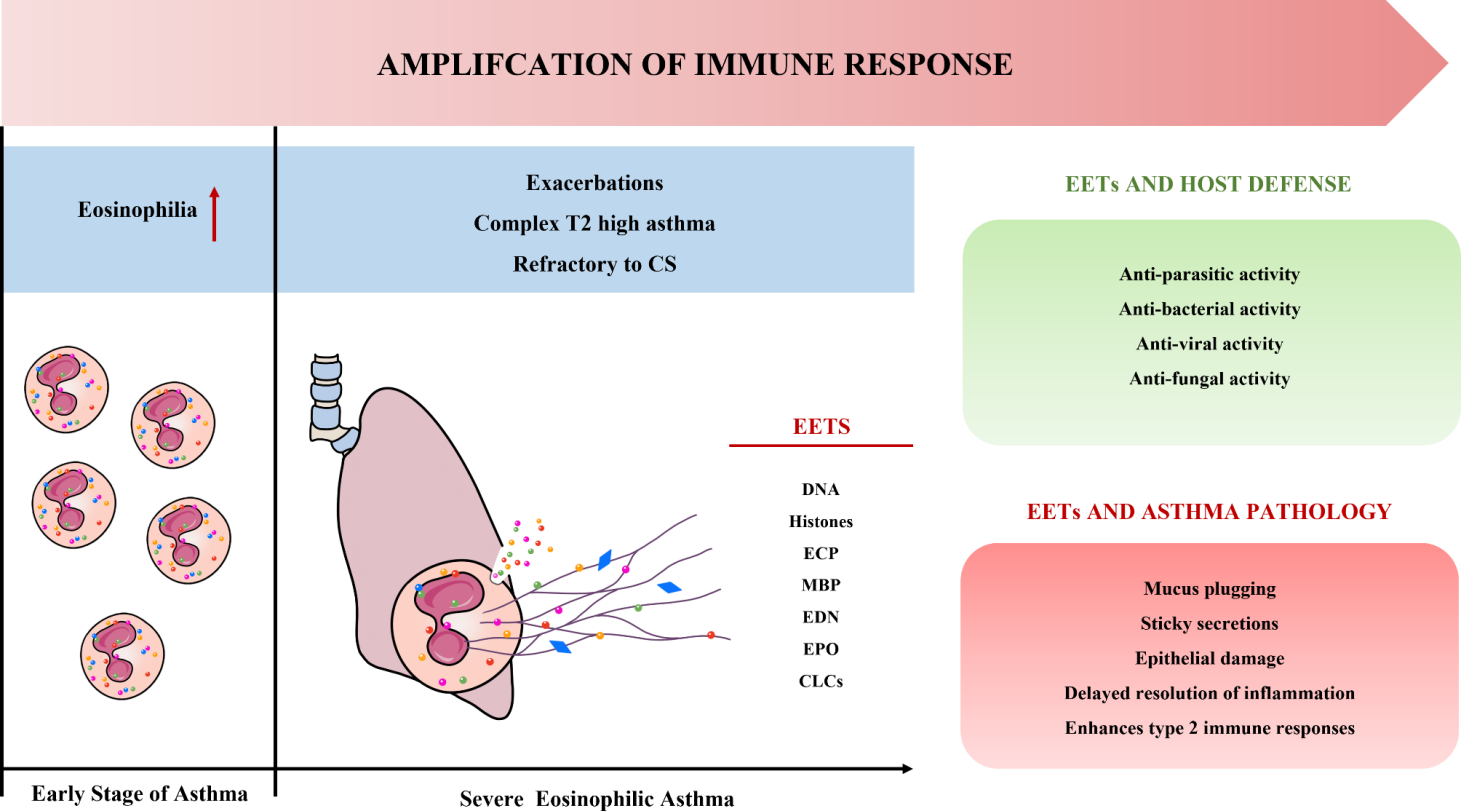
**The double-edged sword effect of EETs.** EETs are reticular structures composed of DNA, histones, and granulins that are formed by eosinophils and released into the extracellular compartment, forming one of the components of the innate immune response. Components of EETs play an active role in the control of infections by trapping and destroying microorganisms, including viruses, fungi, bacteria, and protozoan pathogens. Although EETs can protect the host from microorganisms, excessive EETs can be harmful to the host. While the early stages of asthma are characterized by an increase in eosinophils, in the severe stage of asthma, activated eosinophils can release excessive amounts of EETs, causing airway epithelial damage and CLC production, making sputum more difficult to cough up, in addition to exacerbating the type 2 inflammatory response by interacting with other immune cells. T2: Type 2; CS: Corticosteroids; EETs: Eosinophil extracellular traps; ECP: Eosinophil cationic protein; MBP: Major basic protein; EDN: Eosinophil-derived neurotoxin; EPO: Eosinophil peroxidase; CLCs: Charcot-Leyden crystals

These two phases of treatment should be considered complementary rather than independent. It is important to note that just as the formation and release of NETs is a double-edged sword [45], this feature also exists in EETs (Fig. 2). For example, the released granulocytes restrict this action to specific regions by wrapping granulocyte proteases in the DNA network, which may lead to a strong immune response causing organ dysfunction and even organ failure [8].

**Fig. 3:**
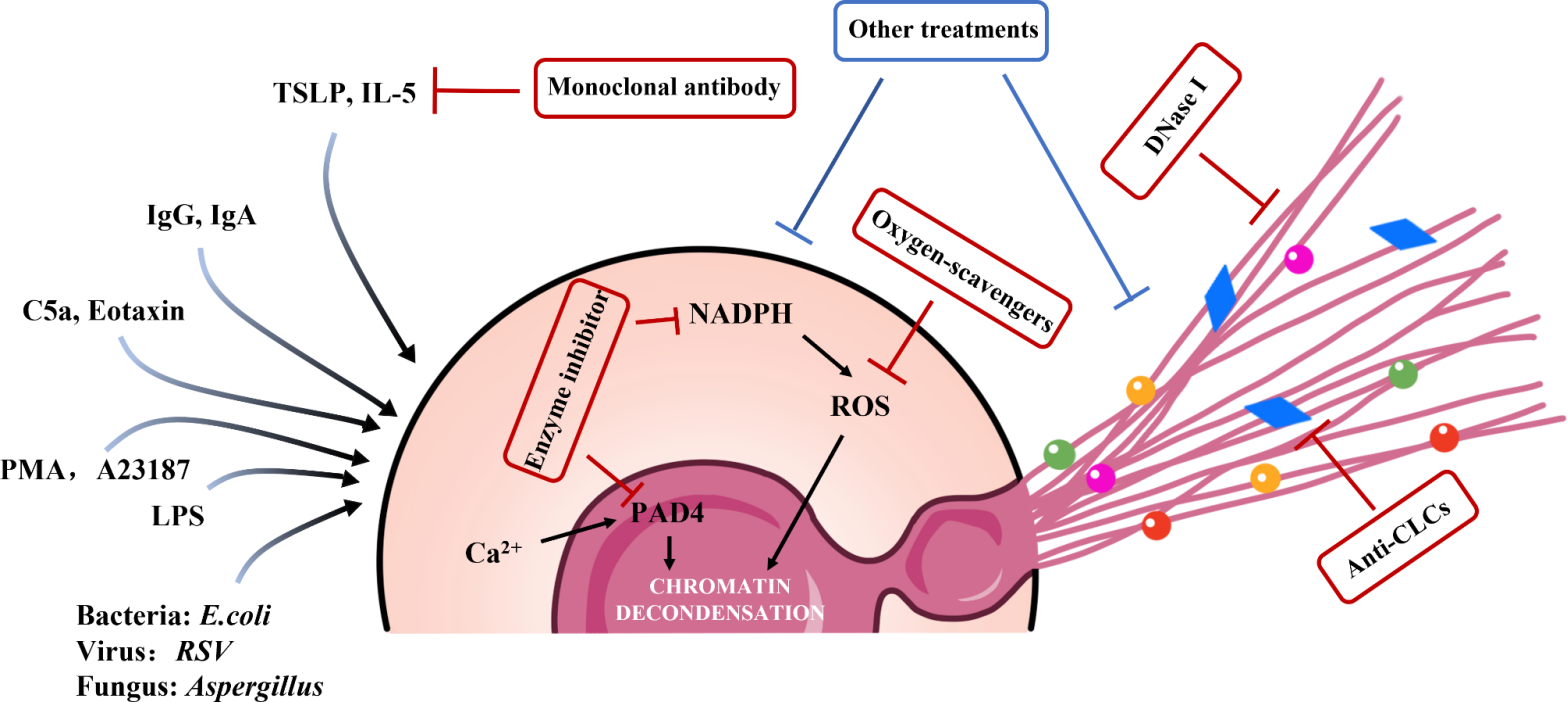
**Therapeutic potential drugs targeting a step in the release and formation of EETs.** Many stimuli can induce eosinophil activation and the release of EETs, so therapeutic strategies for EETs need to be evaluated and selected on an individual basis. E.coli: Escherichia coli; RSV: Respiratory Syncytial Virus; PMA: Phorbol 12-myristate 13-acetate; LPS: lipopolysaccharide; C5a: Complement factor 5a; Ig: Immunoglobulin; TSLP: Thymic stromal lymphopoietin; IL-5: Interleukin 5; NADPH: Nicotinamide adenine dinucleotide phosphate; PAD4: Peptidyl arginine deaminase 4; ROS: Reactive oxygen species; DNaseI: Deoxyribonuclease I; CLCs: Charcot-Leyden crystals

Therefore, the feasibility of targeting systemic EETs for degradation therapy remains uncertain, and further studies are needed to evaluate the timing, monitoring metrics, and potential side effects of such therapy. Although several studies have explored the therapeutic targets of ETs, the structural characteristics of ETs currently make it difficult to find an ideal therapeutic strategy [72]. In the following section, we provide a brief overview of potential future drugs that could be used to treat EETs based on the available studies in the literature and their relevance to directly targeting EETs (Table 1; Fig. 3).

**Table 1:** Drugs that target a phase of the process of eosinophil extracellular traps (EETs) release

Therapy	First author [ref.]	Study description	Related Findings
**DNase treatments**	Lu [10]	Mouse model	DNase I treatment ameliorates asthma in mice
	Cunha [75]	Mouse model	rhDNase decreased significantly airway resistance, EETs formation, and globet cells hyperplasia
	Chia [76] Hull [77] Harrison [78]	Case report	Successful weaning from mechanical ventilation and ongoing recovery
	Boogaard [79]	121 children with moderate to severe worsening asthma were randomly assigned to receive a single 5 mg dose of nebulized rhDNase or a second dose of bronchodilator followed by placebo	Compared to the placebo group, rhDNase did not significantly improve patients’ asthma scores, duration of oxygenation, or number of bronchodilator treatments within 24 h
	Silverman [80]	50 patients aged 18–55 years with FEV1 < 60% and symptomatic asthma were randomly assigned to the 2.5 mg, 5.0 mg, 7.5 mg, or placebo treatment groups	Compared to the placebo group, rhDNase failed to significantly improve FEV1%
	Krug [87]	40 patients aged 18–64 years with mild asthma were randomly assigned to either the 10 mg SB010 or placebo treatment group	Treatment with SB010 significantly attenuated both late and early asthmatic responses after allergen provocation in patients with allergic asthma
**Anti-TSLP antibody**	Choi [11]	Mouse model	EET-mediated airway inflammation in OVA-challenged mice resulted in significantly increased airway hyperresponsiveness and levels of type 2 cytokines in BALF. Treatment with anti-IL33 and anti-TSLP antibodies significantly reduced AHR
	NCT05280418	Thirty patients > 18 years of age with moderate or severe asthma were randomly assigned to the tespilizumab 210 mg subcutaneous injection every 4 weeks or placebo treatment group.	On-going
**Anti-IL-5 antibody**	Sasaki [95] Masaki [96]	Case report	Benralizumab reduces the expression levels of EETs
**PAD4 inhibitors**	Sim [46]	Purified eosinophils isolated from human peripheral blood	EETs formation induced by PMA, A23187 and its activated platelets can be significantly inhibited by the PAD4 inhibitor GSK484
	Kim [102]	Purified eosinophils isolated from human peripheral blood	LysoPS-mediated EETs formation is partially blocked by the PAD4 inhibitor GSK484
	Barroso [103]	Purified eosinophils isolated from human peripheral blood	Aspergillus fumigatus-induced EETs release occurs in a mechanism independent of PAD4 histone guanylation, and the PAD4 inhibitor GSK484 fails to inhibit Aspergillus fumigatus-mediated release of EETs
**NADPH/ROS inhibitors**	Yousefi [32]	Purified eosinophils isolated from human peripheral blood	DNA release can be detected within 5 min of stimulation of eosinophils with C5a or LPS, reaching maximum levels after 20 min, and the effect can be blocked by inhibitors of reactive oxygen species production
	Ueki [38]	Purified eosinophils isolated from human peripheral blood	IgG, IgA, PAF containing IL-5 or GM-CSF, and non-physiological stimulants, calcium carrier A23187 and PMA can cause EETosis, and this effect can be inhibited by DPI
	Sim [46]	Purified eosinophils isolated from human peripheral blood	PMA-induced EETs formation was completely inhibited by DPI, and A23187-induced EETs formation was partially inhibited by DPI. In contrast, conditioned medium and pellet-formed EETs from A23187-activated platelet cultures were completely insensitive to DPI
	Silveira [117]	Mouse model	DPI and NAC treatment reduced EPO, goblet cell proliferation, pro-inflammatory cytokines, NFκB p65 immune content, and lung oxidative stress, and decreased the release of EETs in the airways
	Kim [102]	Purified eosinophils isolated from human peripheral blood	LysoPS-induced EETs are not affected by DPI
**SP-D treatment**	Yousefi [143]	Purified eosinophils isolated from human and mouse peripheral blood	SP-D binds directly to membranes and inhibits human and murine eosinophil-forming EETs in a concentration- and carbohydrate-dependent manner
**cysLT synthase/receptor inhibitor**	Cunha [137]	Mouse model	MK-886 or/and MK-571 treatment reduced cysLT production or inhibited cysLT1 receptors and reduced EETs formation in BALF, respectively
**Autophagy Inhibitors**	Silveira [145]	Mouse model	3-Methyladenine treatment reduced the number of eosinophils, EPO activity, goblet cell proliferation, pro-inflammatory cytokines and NFκB p65 immune content in the lung, improved oxidative stress, mitochondrial energy metabolism and Na + and K+-ATPase activity, and reduced EETs formation in the airways
**Anti-TIMP-1 antibody**	Cao [149]	Cellular model	TIMP-1 directly activates eosinophils and induces EET release. Anti-TIMP-1 antibody inhibits EET release.
**miR-155 Inhibitor**	Kim [151]	Mouse model	miR-155 contributes to the extracellular release of dsDNA and exacerbates allergic lung inflammation. Mixed neutrophil/eosinophil asthma lung inflammation and severe airway hyperresponsiveness can be reduced with miR-155 inhibitors.

DNase I: Deoxyribonuclease I; rhDNase: recombinant human deoxyribonuclease; EETs: Eosinophil extracellular traps; FEV1%: Forced Expiratory Volume in one second percentage; BALF: Bronchoalveolar Lavage Fluid; IL: interleukin; TSLP: Thymic Stromal Lymphopoietin; AHR: Airway Hyperresponsiveness; PMA: Phorbol 12-Myristate 13-Acetate; PAD4: Peptidyl arginine deaminase 4; LysoPS: Lysophosphatidylserine; C5a: Complement factor 5a; LPS: Lipopolysaccharide; IgG: Immunoglobulin G; IgA: Immunoglobulin A; PAF: Platelet-activating factor; GM-CSF: Granulocyte-Macrophage Colony-Stimulating Factor; DPI : Diphenyliodonium chloride; NAC: N-acetylcysteine; EPO: Eosinophil Peroxidase; SP: Surfactant-specific proteins; cysLT: Cysteinyl leukotriene. TIMP-1: Tissue inhibitor of metalloproteinase-1; miR-155: microRNA-155; dsDNA: double-stranded deoxyribonucleic acid

**Table 2:** The effect of the eosinophil extracellular traps (EETs) on other types of cells

Interacting cells	Study description	Related Findings	Ref
Airway epithelial cell	A549, BEAS-2B, human primary small airway epithelial cells and mouse model	EETs caused dose-dependent changes in the morphology and density of A549 cells, leading to more than 10% cell detachment and increased epithelial permeability. Moreover, EETs significantly increased the release of epithelium-derived cytokines, inducing a type 2 immune response	[11, 93]
PNECs	PAD4 ^*−/−*^ mouse model and H146 cells	EET induces PNEC to secrete neuropeptides and neurotransmitters, exacerbating asthma inflammation.	[10]
ILC2s	EETs-stimulated mouse model l	Altered activation status of ILC2 in lung tissue of EET-treated mice and increased proportion of IL-5 or IL-13 producing ILC2 in the lung.	[11]
Eosinophils	Purified eosinophils isolated from human peripheral blood	Consistent with PMA stimulation, the induction of EETs led to morphological changes in eosinophils and significantly increased eosinophil degranulation and ROS production. However, both effects were found to be weaker compared to the effects of PMA stimulation.	[93]
Macrophages	primary human monocyte-derived macrophages differentiated from CD14 + monocytes	CLCs, a component of EETs, can release the pro-inflammatory cytokine IL-1β upon induction of phagocytosis by primary human macrophages in vitro	[22]

EETs: Eosinophil extracellular traps; PNECs: Pulmonary neuroendocrine cells; PAD4: Peptidyl arginine deaminase 4; ILC2s: Group 2 innate lymphoid cells; IL:interleukin; PMA: Phorbol 12-myristate 13-acetate; ROS: Reactive oxygen species; CLCs: Charcot-Leyden crystals

### Targeting the degradation of the chromatin structure of EETs: deoxyribonuclease I (DNase I)

DNase I is an enzyme that can cleave single-stranded DNA, double-stranded DNA, and chromatin. Its recombinant form has been shown to hydrolyze extracellular DNA in sputum and reduce its viscosity, thereby enhancing the clearance of respiratory secretions [73, 74]. Clinically, recombinant human DNase I has been used to treat patients with cystic fibrosis (CF) lung disease, and several studies have evaluated its potential use in asthma.

In the area of ETs, DNase I is widely used as a therapeutic intervention in animal models of allergic and non-allergic diseases. It effectively degrades extracellular DNA traps released by neutrophils and eosinophils, leading to a reduction in associated pathological changes. In mouse models, DNase I treatment has been shown to significantly reduce the production of EETs, thereby attenuating lung inflammatory cell infiltration, and goblet cell proliferation, and mucus production [10, 75]. It also improves airway resistance and lung injury while significantly reversing EET-mediated neuroimmune pathways, thereby slowing the progression of asthma[10]. However, these beneficial effects have shown greater inconsistency in clinical trials in asthma.

Several case reports have suggested that nebulized or direct endotracheal infusion of recombinant human deoxyribonuclease (rhDNase) can significantly improve ventilation and gas exchange in adult or pediatric patients with severe asthma who do not respond to conventional treatment regimens [76–78]. However, the efficacy of DNase has been questioned in two cohort studies: In a previously published multicenter, randomized, double-blind, controlled clinical trial of 121 children admitted to the emergency department with moderate to severe asthma exacerbations, nebulized 5 mg rhDNase failed to significantly improve asthma outcomes in terms of duration of oxygen and bronchodilator treatment during the first 24 h compared to the placebo group [79]. In another prospective study, a total of 50 non-intubated adult ED asthmatics were enrolled to evaluate whether nebulization of various doses of rhDNase improved lung function and disease severity in acute severe asthma in patients who did not respond to conventional nebulized therapy. Results showed that overall, rhDNase treatment did not show a significant benefit [80].

There are several explanations for the inconsistency of rhDNAse in clinical trials and basic research. First, we note that both clinical trials included patients with moderate or severe asthma exacerbations requiring emergency department visits as inclusion criteria. This may be based on early case reports showing a potential benefit of rhDNase in such patients. As one of the therapeutic agents for cystic fibrosis, rhDNase reduces mucus viscosity and adhesion by cleaving extracellular DNA [81]. Mucus obstruction is one of the pathophysiological features of acute asthma [82, 83]. Thus, the use of mucolytics may reverse airway obstruction in asthma and provide clinical benefit.

However, both Silverman and Boogaard expressed doubts in their studies about whether the degradation of DNA content in asthmatic mucus is sufficient to reverse mucus plugging [79, 80]. Unlike the mucus of patients with chronic cystic fibrosis, the level of DNA content in mucus, even in patients with worsening asthma, is only 3–16% of the levels in chronic cystic fibrosis [84, 85]. This may imply that the use of rhDNase alone may not completely reverse the degree of mucus obstruction in asthmatic patients.

Second, the timing of rhDNase intervention may influence the prognosis of asthma. In most studies of OVA mouse models of asthma, the timing of intervention for DNase I was chosen to be during the OVA challenge phase, thus showing that DNase I treatment improves the asthma inflammatory response[10]. However, there were apparent differences with the disease state of patients with moderately severe acute asthma in clinical trials.

Finally, the duration of rhDNase treatment may also influence the asthma treatment effect. Compared to the study by Cunha et al., which showed no effect of rhDNase treatment on inflammatory cells in mouse BALF, the study by Lu et al. observed a similar number of eosinophils in the BALF of DNase I-treated and untreated mice on day 15. However, on day 17, the number of eosinophils in the BALF of DNase I-treated mice was significantly reduced, and the changes in the number of dendritic cells also exhibited similar characteristics [10, 86]. Interestingly, Cunha et al. also demonstrated that rhDNase, in addition to acting as a mucolytic agent, reduced oxidative stress in the lungs of asthmatic mice and exhibited potential antioxidant effects, suggesting a novel mechanism of action for rhDNase.

A promising treatment for asthma is a novel DNA enzyme, SB010 [87]. Phase II clinical trials have demonstrated the efficacy of SB010 in attenuating late and early asthma responses after allergen exposure and in reducing TH2 inflammatory responses in patients with allergic asthma. SB010 can act by cleaving and inactivating GATA3 messenger RNA. However, further studies are needed to determine the potential contribution of rhDNase in the treatment of asthma patients and to assess its efficacy in different asthma populations.

### Inhibiting epithelial-derived Cytokines: Anti-TSLP therapy

TSLP is an epithelial cell-derived cytokine that plays an important role in allergic inflammation by promoting and activating the expression and secretion of Th2 cells, airway dendritic cells, eosinophils, and ILC2 to participate in the innate and acquired immune cascade response [88, 89].

Simon et al. analyzed tissue samples from 18 patients with active eosinophilic esophagitis and found that TSLP was expressed mainly in the apical portion of epithelial cells and correlated significantly with the number of EET-positive eosinophils. This suggests that TSLP may directly activate the production and release of EETs by infiltrating eosinophils [90]. Morshed et al. performed in vitro cellular assays demonstrating that TSLP can induce the release of eosinophil EETs in a concentration- and time-dependent manner and is involved in activating the antimicrobial activity of EETs [44]. In a mouse model of asthma, anti-TSLP antibodies were found to effectively inhibit airway hyperresponsiveness in mice treated with EETs [11].

Therefore, TSLP may play a critical role in mediating the crosstalk between airway epithelial cells and the allergic inflammatory cascade response [88, 91]. A 16-week randomized controlled trial (NCT05280418) is currently underway to evaluate the effects of tezepelumab on airway structure and function in patients with uncontrolled moderate-to-severe asthma, including EETs as one of the outcome measures. Further real-world studies are eagerly awaited to shed more light on the potential clinical utility of targeting TSLP in asthma therapy.

### Targeting T2 Cytokines: Anti-IL-5 therapy

IL-5 plays a critical role in the regulation of eosinophil proliferation and maturation. It promotes eosinophil survival in tissues by delaying apoptosis and inducing degranulation [92]. In vitro studies have shown that IL-5 can stimulate eosinophils to release EETs in a ROS-dependent manner [32]. Furthermore, in patients with severe eosinophilic asthma, peripheral blood eosinophils exhibit enhanced release of EETs when co-stimulated with IL-5 and LPS [93], suggesting that elevated levels of IL-5 in vivo may play an important role in the pathogenesis of severe eosinophilic asthma [94].

Targeting IL-5 as a therapeutic strategy to reduce asthma exacerbations is well established, and three classes of antibodies targeting IL-5 or IL-5R, including mepolizumab, reslizumab, and benralizumab, have shown promising results in reducing asthma exacerbations and improving disease control in phase 3 clinical trials. However, there are only scattered case reports suggesting that benralizumab may be an effective biologic agent to attenuate eosinophilic disease in EETosis [95, 96], lacking support from stronger levels of evidence studies. Further future studies are needed to assess the promise of IL-5 antagonists in EETs with asthma.

### Peptidyl Arginine Deaminase (PAD) 4 inhibitor therapy

PAD4 is a calcium-dependent nuclease that plays a critical role in the formation and release of NETs [97]. PAD4 alters DNA binding to histone by mediating the conversion of specific arginine to citrulline in the histone tails of nucleosomes, which in turn promotes chromatin decondensation and ultimately the release of NETs [98]. Inhibition of PAD4 reduces the number of neutrophils that undergo histone citrullination, and PAD4-deficient mice have been shown to be unable to form citrullinated histone H3 and NETs [99, 100]. EETs formation, like neutrophil extracellular trap cell death (NETosis), is also dependent on PAD4 activation [24, 39, 101].

Studies have shown that a PAD4 inhibitor blocks the formation of EETs mediated by lysophosphatidylserine (LysoPS), PMA, A23187 and its stimulated platelets [46, 102]. However, the necessity of PAD4 for EETs formation remains controversial. Barroso et al. reported that Aspergillus can induce EETs release independently of PAD4 and histone citrullination mechanisms, and a PAD4 inhibitor was unable to inhibit Aspergillus fumigatus-mediated EETs formation [103]. This suggests that the requirement of PAD4 for EETosis may vary depending on the stimulus for EETosis.

Most of the current PAD inhibitors are biologically inefficient, which makes their clinical application relatively slow [104]. F- and Cl-amidine are currently the most effective irreversible pan-PAD inhibitors, which have been shown to be active against PAD4 in vitro and in vivo and to significantly reduce the production of NETs [105–109]. GSK199 and GSK484 are the most widely used reversible PAD4 inhibitors. Compared to Cl-amidine, specific PAD4 inhibitors have less off-target effects and drug toxicity [104], and also significantly reduce the level of NETs [110–112].

Although PAD inhibitors have shown significant efficacy in animal models of human disease [104], there are a limited number of studies on PAD4 inhibitors in asthma. Chen and colleagues observed that treatment with simvastatin significantly reduced the expression levels of PAD4 and NETosis in both asthmatic mice and LPS-induced HL-60-differentiated neutrophil-like cells [113]. They found that simvastatin significantly reduced the expression levels of PAD4 and NETosis in these mice. This suggests that simvastatin may be a promising new strategy for targeting PAD4 to improve extracellular trap release in asthma, but the efficacy of its targeting of EETosis is not known, and further studies are needed to clarify.

### Nicotinamide Adenine Dinucleotide phosphate (NADPH)/ROS inhibitor therapy

Excessive eosinophil and neutrophil infiltration in asthma result in elevated ROS levels with impaired antioxidant response, leading to oxidative stress and promoting airway inflammation and hyperresponsiveness [114]. Studies have shown that activation of NADPH oxidase mediated the production of ROS and is one of the key mechanisms of EETosis in eosinophils [8, 38]. Yousefi and colleagues reported that stimulation of human peripheral blood eosinophils with IL-5 or interferon-gamma (IFN-γ) combined with LPS, C5a, or eosinophil chemokines leads to a rapid release of extracellular DNA from eosinophils. This effect is dependent on the generation of ROS and can be blocked by ROS inhibitors [32].

Diphenylammonium chloride (DPI) and N-acetylcysteine (NAC) are NADPH oxidase inhibitors and ROS scavengers, respectively, both of which reduce intracellular ROS levels [115, 116].Silveira et al. found that DPI and NAC treatment reduced the number of inflammatory cells and ROS levels in the lungs of OVA-challenged mice, in addition to reducing airway levels of released EETs [117]. However, as mentioned above, not all stimuli are dependent on the PAD4 mechanism, and the necessity of NADPH enzymes for EETs formation is controversial. Studies have shown that while DPI can completely or partially inhibit A23187-mediated EETs release [38, 46, 102], it cannot inhibit EETs release mediated by A23187-stimulated platelets and lysoPS [46, 102]. These findings suggest that there are multiple pathways to initiate the formation of EETs and that therapeutic strategies for EETs need to be evaluated and selected on an individual basis.

Despite the importance of ROS in asthma pathogenesis, antioxidant-based therapeutic strategies have yielded limited success in asthma management. While NAC has demonstrated efficacy in animal models of asthma and small clinical trials, its use outside of standard therapy has not demonstrated significant impact. Antioxidant compounds including superoxide dismutase mimetics, polyphenols, and small molecule nuclear factor erythroid 2-related factor 2 (Nrf2) activators also lack extensive clinical validation. NADPH oxidase inhibitors and mitochondria-targeted antioxidants represent novel targeting approaches currently under clinical investigation for certain diseases, but have not been fully explored in asthma and require further investigation in future studies [114].

Mono-n-butyl phthalate (MnBP) is a common endocrine disrupting chemical, mainly derived from external environmental intake, which has been shown to be associated with a high risk of asthma [118–120]. Quoc et al. found that MnBP induces ROS production and promotes the release of EETs in human eosinophils and mouse lung tissue and BALF. They also found that vitamin E treatment can control the Nrf2-ROS-EET pathway by inhibiting ROS, which may be a potential treatment for MnBP-induced eosinophilic airway inflammation [121].

Studies suggest that vitamin E may influence the development of allergy and reactivity to allergens early in life, and several prospective, observational, and randomized prevention studies have been conducted to assess the relationship between vitamin E intake and asthma risk, but the results of these studies are inconsistent [122–124]. Allen et al. examined the association between vitamins (measured by dietary intake or serum levels) and asthma in participants from 24 prospective cohort and case-control studies and reported that vitamin E intake did not appear to be associated with asthma status [125]. This may be related to the composition of vitamin E, an oxidant consisting of eight fat-soluble compounds, including four tocopherols (d-α-, d-β-, d-γ-, and d-δ-tocopherols) and four tocotrienols (d-α-, d-β-, d-γ-, and d-δ-tocotrienols) [126]. Among them, α- and γ-tocopherols have opposite functions in the regulation of allergic inflammation and disease progression, with α-tocopherol subtype being anti-inflammatory and γ-tocopherol subtype being pro-inflammatory, and differences in the doses of these two subtypes in dietary supplements may partially explain the differences in the results of different clinical trials, and further studies are needed to evaluate them in the future [122–124].

### Exogenous complementary therapy: pulmonary surfactant

Pulmonary surfactant, a vital lipoprotein complex located in the lung wall, is synthesized primarily by alveolar type 2 cells. It was originally thought to be a physical factor that coordinates various biological processes in the body. However, recent studies have shown that it also plays a critical role in pulmonary innate and adaptive immunity [127–129]. There are currently six lung surfactant-specific proteins (SP), including SP-A, SP-B, SP-C, SP-D, SP-G, and SP-H [130, 131].

SP-A and SP-D play important roles in asthma [128, 129]. Studies have shown that SP-A and SP-D levels are lower in asthmatics than in healthy subjects and are inversely correlated with asthma severity [132, 133], possibly due to their role as scavengers in a chronically inflammatory environment that constantly depletes them. In addition, chronic inflammation can cause damage to alveolar type 2 cells, resulting in reduced secretion [127], and a study by Yousefi et al. found that SP-D inhibited eosinophil activation and blocked the formation of EETs. This effect is completely lost when SP-D is S-nitrosylated [134]. Furthermore, the integrity of the SP-D structure is more severely compromised in BALF and serum samples from patients with severe asthma, as observed in a study by Mackay et al. [132]. This may partially explain the decreased levels of SP-D and increased levels of EETs in patients with severe asthma.

Exogenous pulmonary surfactant was tried in the 1960s and found to be effective in premature infants with respiratory distress syndrome. It has since been used in children with pneumonia, acute lung injury, or acute respiratory distress syndrome. However, it has been observed that pulmonary surfactant administration is not beneficial in adults with acute lung injury or acute respiratory distress syndrome [128].

Despite the potential benefits of pulmonary surfactants in the treatment of respiratory diseases, their clinical applications still face significant challenges. One of these challenges is the difficulty in developing formulations that combine stability and solubility, particularly in the case of SP-D due to its complex structural properties. In addition, immune response and large-scale mass production pose further challenges to widespread use of pulmonary surfactants [128, 135]. To address these issues, smaller human recombinant trimeric fragments of SP-A and SP-D have been successfully developed [136]. However, the efficacy of SP-D supplementation in the treatment of asthma and other respiratory diseases is still not fully understood. Nevertheless, the use of exogenous supplementation with recombinant SP-A and SP-D may represent a promising new direction in the treatment of inflammatory respiratory diseases [127].

### Other treatments

Cysteinyl leukotriene (cysLT) induces eosinophil inflammation, where activation of the cysLT1 receptor induces bronchoconstriction, airway edema, and mucus secretion in asthmatic patients. Cunha et al. found that the cysLT synthase inhibitor MK-886 and the cysLT1 receptor antagonist MK-571 reduced airway EETs formation in OVA-challenged mice [137]. Both MK-886 and MK-571 have been tested in asthma patients, and studies have shown that they reduce asthma symptoms, β2 agonist dose, and bronchoconstriction induced by exposure to allergens, exercise, aspirin, and cold air [138–143]. These studies offer the possibility of developing appropriate targeted therapies for EETs, but further studies are needed to confirm this.

Autophagy plays a crucial role in inflammatory diseases, and its role is closely related to the severity of asthma through eosinophil inflammation [144]. Silveira et al. found that treatment with the autophagy inhibitor 3-methyladenine reduced the formation of airway EETs in OVA-challenged mice, thereby reducing the airway immunopathological manifestations of asthma[145]. There are no clinical trials of autophagy inhibitors in asthma patients, and further studies are needed to assess their future therapeutic potential.

Matrix metalloproteinase (MMP)/tissue inhibitor of metalloproteinase (TIMP) interactions play a key role in the production, degradation, and structural alteration of extracellular matrix as an important part of airway remodeling in chronic asthma [146]. TIMP-1 is a major inhibitor of MMP-9, and it possesses fibrotic properties, promoting the growth of fibroblasts and myofibroblasts [147]. The level of TIMP-1 was found to be significantly higher in asthmatics than in normal subjects and negatively correlated with FEV1% and FVC [148]. The CD63 receptor, a key receptor for TIMP-1, is highly expressed on eosinophils in patients with severe asthma. Cao et al. found that TIMP-1 induces eosinophil activation and EETs release through the CD63/PI3K signaling axis. Interestingly, only anti-CD63 treatment showed the ability to reduce the level of EETs compared to dexamethasone treatment, which may suggest the value of anti-TIMP-1 treatment in addressing steroid resistance [149].

In mixed granulocytic asthma, there is a lack of more effective, specific therapeutic options. MicroRNAs are responsible for the post-transcriptional regulation of genes, of which miR-55 is thought to play a key role in TH2-mediated eosinophil inflammation [150]. miR-155 was found by Kim et al. to be an early regulator of host dsDNA and ETosis release in severe asthma, which may contribute to airway damage and mixed granulocyte inflammation by promoting dsDNA release. The use of miR-155 inhibitors may reduce lung inflammation and severe airway hyperresponsiveness in mixed neutrophilic/eosinophilic asthma, but it is worth noting that the timing of dosing may significantly affect the outcome [151].

## Conclusion and perspectives

EETs not only participate in the pathogenesis of asthma but also in many other autoimmune and inflammatory diseases, including chronic obstructive pulmonary disease, sepsis, and vascular disease. Despite numerous studies investigating the formation and function of EETs, the specific mechanisms of EETs regulation remain unknown. EETosis proceeds through a specific sequence of events (Fig. 1). Most of the current literature have investigated the sources of stimulation and effective inhibitors of EETosis. Further investigation of how they affect the cellular events of EETosis may help us understand the underlying mechanisms and identify new therapeutic targets. EETosis, being a ‘double-edged sword’, has primarily been focused on its negative effects in the field of asthma, while the value of its positive effects has been less explored. Furthermore, as one of the physiologically released substances, there is a lack of large-scale studies to define the range of normal levels. Objective reference values would be valuable for clinicians to assess guidelines for therapeutic intervention. Of all the issues raised in this review, several may deserve special attention as they can significantly contribute to our understanding of the cell biology of EETosis, its biophysics, and its role in the field of asthma (Table 3).

**Table 3:** Future issues

1.	How is EETosis initiated?
2.	What is the mechanism of vital EETosis?
3.	Improvement in various types of asthma with rhDNase treatment
4.	The role of EETs in viral-induced asthma exacerbations
5.	It is unclear whether the formation of EETs in asthma is regulated by other cells or molecules. Understanding these regulatory mechanisms could help us better understand the process of asthma exacerbations and provide targets for the development of new therapeutic approaches.
6.	The distribution of EETs in patients with asthma is not fully understood. Further studies may reveal how it varies by type and severity of asthma and how it relates to asthma symptoms and response to treatment.
7.	The potential role of EETs in the treatment of asthma requires further investigation. Some studies suggest that inhibition of EETs formation and function may be beneficial in reducing asthma symptoms and ameliorating inflammation. However, no specific therapeutic strategy targeting EETs has been widely adopted.

## Data Availability

Not applicable.

## Abbreviations

NETs: Neutrophil extracellular traps
EETosis: Eosinophil extracellular trap cell death
EETs: Eosinophil extracellular traps
OVA: ovalbumin
EDGPs: Eosinophil-derived granule proteins
EPO: Eosinophil peroxidase
MBP: Major basic protein
ECP: Eosinophil cationic protein
EDN: Eosinophil-derived neurotoxin
Gal10: Galectin-10
CLCs: Charcot-Leyden crystals
Ig: Immunoglobulin
ETosis: Extracellular trap cell death
ETs: Extracellular traps
ROS: Reactive oxygen species
IL: interleukin
LPS: lipopolysaccharide
C5a: Complement factor 5a
TSLP: Thymic stromal lymphopoietin
Ig: Immunoglobulin
PMA: Phorbol 12-myristate 13-acetate
GM-CSF: Granulocyte-macrophage colony-stimulating factor
CXCL-1: Chemokine ligand 1
BALF: Bronchoalveolar lavage fluid
CK18: Cytokeratin 18
FEV1: Forced expiratory volume in one second
ILC2: Group 2 innate lymphoid cells
dsDNA: Double-stranded DNA
citH3: Citrullinated histone H3
cfDNA: Circulating free DNA
EGPA: Eosinophilic granulomatosis with polyangiitis
CRS: Chronic rhinosinusitis
DNase I: Deoxyribonuclease I
rhDNase: recombinant human deoxyribonuclease
CF: Cystic Fibrosis
PAD: Peptidyl arginine deaminase
NETosis: Neutrophil extracellular trap cell death
lysoPS: Lysophosphatidylserine
NADPH: Nicotinamide adenine dinucleotide phosphate
DPI: Diphenyliodonium chloride
IFN-γ: Interferon-gamma
NAC: N-acetylcysteine
Nrf2: nuclear factor erythroid 2-related factor 2
MnBP: Mono-n-butyl phthalate
SP: Surfactant-specific proteins
cysLT: Cysteinyl leukotriene
